# Resmetirom-eligible population among US adults: An estimation analysis based on NHANES 2017–March 2020

**DOI:** 10.1097/HC9.0000000000000755

**Published:** 2025-06-19

**Authors:** Phuc Le, Eda Kaya, Anh Phan, Yusuf Yilmaz, Naim Alkhouri

**Affiliations:** 1 Cleveland Clinic, Cleveland, Ohio, USA; 2 Department of Medicine, Ruhr University Bochum, University Hospital, Knappschaftskrankenhaus Bochum, Bochum, Germany; 3 Department of Hepatology, The Global NASH Council, Washington, District of Columbia, USA; 4 The Charter School of Wilmington, Wilmington, Delaware, USA; 5 Department of Gastroenterology, School of Medicine, Recep Tayyip Erdogan University, Rize, Türkiye; 6 Department of Hepatology, Arizona Liver Health, Chandler, Arizona, USA

**Keywords:** fibrosis, metabolic dysfunction–associated steatohepatitis, metabolic dysfunction–associated steatotic liver disease, noninvasive test, resmetirom

## Abstract

**Background:**

Resmetirom received FDA approval for treating adults with noncirrhotic metabolic dysfunction–associated steatohepatitis (MASH) and moderate-to-advanced liver fibrosis (stages F2–F3). Here, we sought to estimate the eligible U.S. adult population for resmetirom therapy, with secondary analysis focusing on individuals with type 2 diabetes mellitus (T2DM).

**Methods:**

A cross-sectional analysis was conducted using data from the National Health and Nutrition Examination Survey (NHANES) 2017–March 2020 cycle. Two eligibility scenarios were examined: a liberal scenario requiring ALT >17 U/L for women or >20 U/L for men, controlled attenuation parameter (CAP) >280 dB/m, and liver stiffness measurement (LSM) >8 kPa; and a restrictive scenario requiring ALT >30 U/L for both sexes, CAP >280 dB/m, and LSM >10 kPa. The analysis incorporated sampling weights to generate nationally representative estimates.

**Results:**

The study cohort included 7244 adults (mean age 49.08 y, 49.9% male) with a mean BMI of 29.61 kg/m², mean CAP 263.35 dB/m, and mean LSM 5.8 kPa. An estimated 8.3 million (95% CI: 6.6–9.9 million) adults met the liberal eligibility criteria, while 2.3 million (95% CI: 1.4–3.2 million) met the restrictive criteria. Patients meeting restrictive criteria were predominantly male (76.2% vs. 59.9%) and younger (mean age 46.7 vs. 48.3 y), with similar BMI (38.6 vs. 38.1 kg/m²). Among adults with T2DM, 3.5 million (95% CI: 2.4–4.5 million; 12.2%) met liberal, whereas 0.85 million (95% CI: 0.5–1.2 million; 3.0%) restrictive criteria.

**Conclusions:**

A substantial proportion of U.S. adults meet eligibility criteria for resmetirom treatment, with estimates varying significantly based on the stringency of selection criteria.

## STUDY HIGHLIGHTS

**What is known?**

- Resmetirom was recently approved by the Food and Drug Administration conditionally in the United States owing to positive results in the improvement of steatohepatitis and fibrosis.

**What is new here?**

- 4% (95% CI 3.2%–4.8%) or 8.3 (6.6–9.9) million US adults would meet the liberal eligibility criteria for resmetirom treatment.
- When applying the more stringent criteria, an estimate of 1.1% (95% CI 0.7%–1.5%) or 2.3 (1.4–3.2) million US adults would be eligible for treatment.
- Strategic planning and support will be needed to mitigate the potential financial burden and ensure access for all eligible patients.

## INTRODUCTION

Metabolic dysfunction–associated steatotic liver disease (MASLD) is defined by the presence of hepatic steatosis accompanied by evidence of metabolic risk factors such as obesity and type 2 diabetes (T2DM). This definition was established and accepted by an international consensus in 2023, following comprehensive deliberation and debate.^1^ The previous term, nonalcoholic fatty liver disease (NAFLD), was superseded due to its limitations in accurately reflecting the disease’s underlying pathophysiology. Notably, recent studies have demonstrated that MASLD and NAFLD encompass virtually the same population, suggesting that the terms could be used interchangeably.^2^

MASLD is currently recognized as the most prevalent chronic liver disease globally, affecting ~38% of the adult population and nearly 10% of children worldwide.^3^,^4^ However, the prevalence of MASLD is significantly higher in individuals with T2DM, reflecting complex bidirectional interactions between these conditions. In addition, epidemiological data have shown that the prevalence of MASLD in individuals with T2DM has recently escalated to 65%, representing a significant 13% increase from 55% over the past 2 decades. The hepatologic implications of this scenario are particularly concerning, as 66% of T2DM patients with MASLD exhibit histologically confirmed metabolic dysfunction–associated steatohepatitis (MASH), while 15% present with advanced fibrosis.^5^ Obesity is another critical determinant in MASLD development, affecting ~70% of individuals with an overweight status and 75% of those with obesity.^6^ Consequently, therapeutic strategies targeting metabolic components, specifically obesity and T2DM, have been identified as promising approaches for MASLD management. Importantly, glucagon-like peptide-1 receptor agonists have demonstrated efficacy in MASH treatment, with several phase III clinical trials currently underway.^7^ Despite the potential of these pharmacological interventions, lifestyle modifications aimed at achieving at least a 5% reduction in body weight remain the cornerstone of MASLD therapy. However, the long-term effectiveness of such lifestyle interventions has been disappointing, with sustained weight loss proving challenging to achieve.^8^,^9^

In a significant advancement, resmetirom—an oral thyroid hormone receptor beta-selective agonist—became the first liver-targeted medication approved by the U.S. Food and Drug Administration (FDA) following the results of the interim analysis from the MAESTRO–NASH trial.^10^ The medication is specifically indicated for patients with noncirrhotic MASH and fibrosis stages F2–F3, with a weight-based dosing protocol: 80 mg once daily for patients weighing <100 kg and 100 mg for those weighing 100 kg or more. While the MAESTRO–NASH trial demonstrated resmetirom’s safety and efficacy in promoting histological improvement in MASLD, further investigation of its long-term effects and potential individual adverse events remains necessary.^11^ The FDA approval introduces a pragmatic approach to patient selection, eliminating the requirement for liver biopsy confirmation. Instead, the diagnostic strategy incorporates a combination of noninvasive tests (NITs), including blood-based biomarkers and vibration-controlled transient elastography (VCTE) by FibroScan (Echosens) evaluation, for assessing and managing MASLD patients. However, the implementation of resmetirom therapy faces significant challenges, primarily due to its substantial annual cost, which may limit accessibility for several patients.^12^ Furthermore, the current lack of comprehensive data regarding the size of the eligible patient population and projected costs for large-scale implementation complicates healthcare planning and resource allocation. To address these knowledge gaps, we sought to estimate the eligible population based on NITs from the MAESTRO–NASH trial, utilizing data from the National Health and Nutrition Examination Survey (NHANES) 2017–March 2020 cycle, which provides a representative sample of the US population.

## METHODS

### Data source

NHANES represents a comprehensive national health assessment program that has been evaluating the health and nutritional status of the US population since the early 1960s. Under the direction of the National Center for Health Statistics (NCHS), a division of the Centers for Disease Control and Prevention (CDC), NHANES combines multiple data collection methods, including personal interviews, physical examinations, laboratory testing, and instrumental diagnostics to create a detailed picture of public health across the nation.^13^ The survey process adheres to the ethical principles outlined in the Declaration of Helsinki, with all participants providing informed consent prior to data collection. Given the public availability and anonymized nature of NHANES data, additional Institutional Review Board approval was not required for our analysis. For this study, we utilized data from the NHANES 2017–March 2020 cycle, which merged with the previous 2017–2018 cycle due to the premature suspension of data collection in March 2020, resulting from the COVID-19 pandemic.

### Definitions and exclusion criteria

**FIGURE 1:**
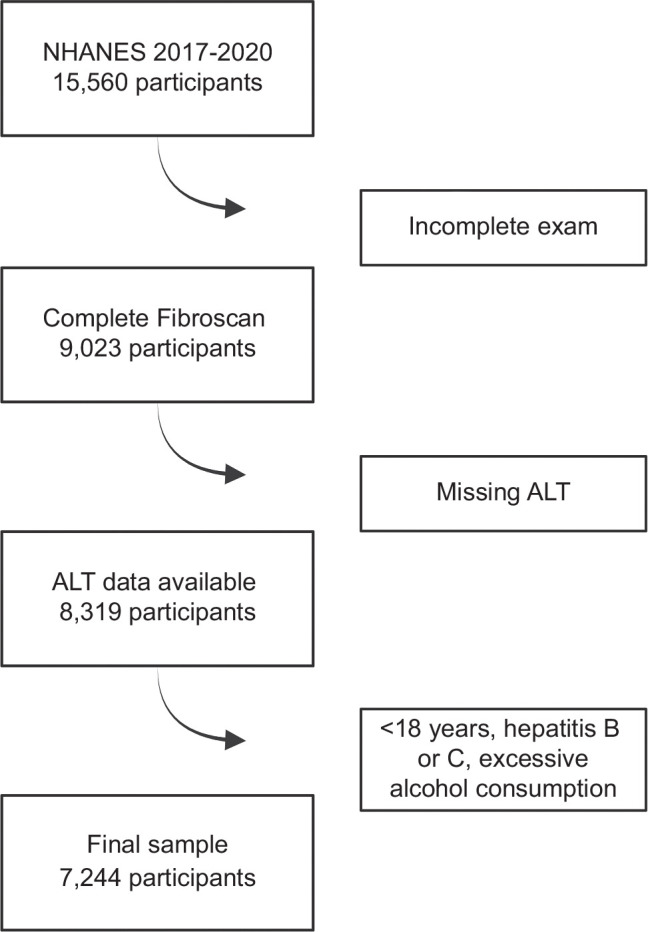
Flowchart of subject selection for inclusion in the current analysis. Abbreviation: NHANES, National Health and Nutrition Examination Survey.

From the NHANES 2017–March 2020 database, we selected adult participants aged 18 years and older for analysis. We applied several exclusion criteria to identify eligible participants: those with documented liver cirrhosis, pregnant women, individuals with viral hepatitis, and those reporting excessive alcohol consumption were excluded. Cirrhosis was identified based on participants ever being told they had liver cirrhosis, a liver stiffness measurement (LSM) >20 kPa, or a platelet count <150 (10^3^ cells/μL). Alcohol consumption was quantified using the Alcohol Use Questionnaire, calculated as (average annual frequency of alcohol intake × average drinks per day)/365. We defined excessive alcohol use as more than 2 drinks daily for men and more than 1 drink daily for women. A controlled attenuation parameter (CAP) threshold of ≥280 dB/m was employed to define hepatic steatosis. Type 2 diabetes mellitus (T2DM) was characterized as either a documented history of T2DM diagnosis, hemoglobin A1c (HbA1c) levels ≥6.5%, fasting glucose >125 mg/dL, or the use of antidiabetic medications. To facilitate further analysis, 2 scenarios were developed based on the pre-screening protocol and laboratory data derived from the MAESTRO–NASH trial^10^ and 2 recent papers that provided guidance on how to use NITs to select patients for resmetirom therapy based on expert panel recommendations.^12^,^14^ In the first liberal scenario based on the October 2024 updates to AASLD Practice Guidance, eligible patients were defined as having a CAP >280 dB/m, LSM >8 kPa, and ALT levels exceeding 17 U/L for women or 20 U/L for men. In contrast, the second setting applied more stringent inclusion criteria based on the paper by Noureddin et al and the MAESTRO–NASH trial, requiring a CAP >280 dB/m, LSM >10 kPa, and ALT >30 U/L for both sexes. The participant selection process and stratification are illustrated in (Figure 1).

### Statistical analysis

The general characteristics of the study population are summarized using descriptive statistics, with results presented as counts and either mean ± SD or median [minimum–maximum]. The primary analysis estimated the population size eligible for liberal and stringent scenarios, while a secondary analysis focused on estimating the population size for these settings specifically among patients with T2DM. The study sample was weighted to be representative of the US civilian non-institutionalized population, and demographic characteristics were reported accordingly. Comparisons were conducted using the chi-square test for categorical variables and the Student *t* test for continuous variables. All analyses applied appropriate survey weights to ensure accuracy and representativeness. Statistical calculations were performed using Stata version 17 (StataCorp LLC), with a 2-sided *p* value of <0.05 considered statistically significant.

## RESULTS

The analysis began with 15,560 participants from the NHANES 2017–2020 survey. Of these, 6537 were excluded due to incomplete FibroScan examinations, 709 due to missing ALT data, and 1075 for meeting one or more of the following criteria: being under 18 years of age, having cirrhosis, excessive alcohol consumption, or a diagnosis of hepatitis B or C, as illustrated in (Figure 1). The final analysis included 7244 adults, with their baseline characteristics summarized in (Table 1). The study population had a mean age of 49 years, and 49.5% were male (n=3585). The prevalence of T2DM was 18.6% (N=1349) and obesity was 43.2% (N=3103). The mean ALT and AST levels were 22.4 and 21.9 U/L, respectively. The FibroScan examinations revealed a mean CAP of 263 dB/m and LSM of 5.8 kPa. Most of the patients were White (34.6%, N=2507).

**TABLE 1:** General characteristics of the study population (N=7244)

	Survey sample (N=7244)				Weighted sample			
	N	Mean, %	95% CI		Sum of weight	Mean/%	95% CI	
Age, mean	7244	49.08	48.66	49.49	206,266,795	47.22	46.03	48.40
Sex								
Female	3659	50.51	49.36	51.66	104,098,794	50.47	48.57	52.36
Male	3585	49.49	48.34	50.64	102,168,001	49.53	47.63	51.42
BMI, mean	7189	29.61	29.44	29.77	206,266,795	29.47	29.11	29.82
Race								
White	2507	34.61	33.51	35.70	129,664,712	62.86	58.02	67.71
Black	1683	23.23	22.26	24.21	34,331,607	16.64	13.38	19.91
Hispanic	1814	25.04	24.04	26.04	21,782,080	10.56	7.79	13.33
Other	1240	17.12	16.25	17.99	20,488,396	9.93	7.85	12.02
Comorbidities								
T2DM	1349	18.62	17.73	19.52	28,374,003	13.76	12.78	14.73
Obesity (BMI ≥30 kg/m^2^ for Caucasians or ≥27 kg/m^2^ for Asian ethnicity)	3103	43.16	42.02	44.31	85,435,344	41.61	39.02	44.19
Laboratory values								
Total bilirubin (mg/dL)	7244	0.46	0.45	0.47	206,266,795	0.47	0.46	0.49
AST (IU/L)	7213	21.94	21.61	22.28	206,266,795	21.92	21.44	22.40
ALT (IU/L)	7244	22.41	21.97	22.85	206,266,795	22.95	22.30	23.61
Albumin (g/dL)	7244	4.08	4.07	4.08	206,266,795	4.12	4.10	4.14
Platelet count (10^3^ cells/μL)	7241	246.64	245.15	248.14	206,266,795	246.92	243.17	250.66
HbA1C (%)	7236	5.82	5.79	5.84	206,266,795	5.65	5.61	5.69
Fibroscan values								
LSM (kPa)	7244	5.8	5.69	5.90	206,266,795	5.70	5.50	5.91
CAP (dB)	7244	263.35	261.91	264.78	206,266,795	263.20	260.51	265.89

*Note*: All estimates were weighted to represent the general US population.

Abbreviations: BMI, body mass index; CAP, controlled attenuation parameter; HbA1c, hemoglobin A1c; LSM, liver stiffness measurement; T2DM, type 2 diabetes mellitus.

### Eligible population for resmetirom therapy based on the liberal criteria

In all, 2801 participants (38.67%) had a CAP value exceeding 280 dB/m, 704 participants (9.72%) had an LSM >8 kPa, and 3158 participants (43.57%) exhibited elevated ALT levels—defined as >17 U/L for females and >20 U/L for males. Notably, a total of 276 individuals (3.81%) met all 3 liberal eligibility criteria, corresponding to an estimated 8.3 million individuals in the US population (4.0%, 95% CI: 3.2–4.8%).

### Eligible population for resmetirom therapy based on the stringent criteria

When the stringent criteria were applied, 75 individuals fulfilled the requirements, representing an estimated 2.3 million eligible individuals (1.1%, 95% CI: 0.7%–1.5%) for resmetirom therapy. The population identified using the stringent criteria was predominantly male (76.2% vs. 59.9%), slightly younger (46.7 vs. 48.3 y), and exhibited similar BMI (38.6 vs. 38.1 kg/m²) and HbA1c levels (6.3% vs. 6.5%). The racial distribution between the 2 groups was also comparable. Also, the group with stringent criteria had higher transaminase levels. Despite utilizing the same CAP threshold, the mean CAP value was higher in the group meeting the stringent criteria (356 vs. 342 dB/m). The LSM value was also slightly higher (12.6 vs. 10.7 kPa).

A comparison of the estimated populations under both scenarios is summarized in (Table 2).

**TABLE 2:** Comparison of estimated populations eligible for resmetirom therapy within the 2 proposed scenarios (N=7244)

	Liberal scenario (CAP ≥280 dB/m+LSM ≥8 kPa+ALT >17 U/L for female and >20 U/L for male)		Stringent scenario (CAP ≥280 dB/m+LSM ≥10 kPa+ALT >30 U/L)	
	N	95% CI	N	95% CI
Patient count				
N	8,252,286	6,642,203–9,862,441	2,287,116	1,387,557–3,186,616
%	4%	3.2%–4.8%	1.1	0.7%–1.5%
Age, mean	48.3	45.0–51.7	46.7	42.0–51.4
Gender, %				
Male	59.9%	52.4%–67.4%	76.2%	66.5%–86.0%
Female	40.1%	32.6%–47.6%	23.8%	14.0%–35.5%
BMI, mean	38.1	36.8–39.3	38.6	36.9–40.3
BMI category, %				
Underweight (≤18 kg/m^2^)	0.0%	NA	0.0%	NA
Normal weight (18.5–25 kg/m^2^)	1.5%	0.3%–2.6%	0.9%	0.6%–1.2%
Overweight (25–30 kg/m^2^)	9.7%	6.0%–13.3%	12.9%	6.3%–19.4%
Obese class I (30–35 kg/m^2^)	20.6%	11.8%–29.4%	15.4%	2.0%–28.7%
Obese classes II and III (≥35 kg/m^2^)	68.3%	57.8%–78.8%	70.9%	56.5%–85.2%
Race				
White	65.3%	57.1%–73.6%	67.6%	56.0%–79.3%
Black	19.8%	14.1%–25.6%	20.8%	12.2%–29.3%
Hispanic	5.5%	2.7%–8.2%	2.9%	1.2%–4.7%
Other	9.4%	4.9%–13.9%	8.7%	2.2%–15.1%
Laboratory values				
Total bilirubin (mg/dL)	0.5	0.4–0.6	0.5	0.4–0.6
AST (IU/L)	31.0	28.4–33.5	41.4	35.1–47.7
ALT (IU/L)	41.8	37.6–46.0	59.1	50.6–67.5
Albumin (g/dL)	4.1	4.0–4.1	4.2	4.1–4.3
Platelet count (10^3^ cells/μL)	259.4	245.4–273.3	257.0	238.4–275.6
HbA1C (%)	6.5	6.2–6.8	6.3	6.0–6.6
Prevalence of diabetes (%)	42.0	32.0–52.0	37.1	25.7–48.6
Fibroscan values				
LSM (kPa)	10.7	10.2–11.2	12.6	11.9–13.4
CAP (dB)	343.0	335.7–350.2	356.2	349.4–363.1

*Note*: All estimates were weighted to represent the general US population.

Abbreviations: BMI, body mass index; CAP, controlled attenuation parameter; HbA1c, hemoglobin A1c; LSM, liver stiffness measurement; T2DM, type 2 diabetes mellitus.

Among the analyzed population, T2DM was identified in 1349 participants (18.62%). Of these, 140 patients (10.38%) met the eligibility criteria for resmetirom under the liberal scenario, whereas 39 participants (2.89%) qualified under the stringent criteria. These figures correspond to estimated populations of 3.5 million and 0.8 million individuals, respectively. The comparison between the 2 scenarios revealed a similar pattern to that observed in the general population (Table 3).

**TABLE 3:** Comparison of estimated populations with T2DM eligible for resmetirom therapy within the 2 proposed scenarios (N=1349)

	Liberal scenario (CAP ≥280 dB/m+LSM ≥8 kPa+ALT >17 U/L for female and >20 U/L for male)		Stringent scenario (CAP ≥280 dB/m+LSM ≥10 kPa+ALT >30 U/L)	
	Mean, %	95% CI	Mean, %	95% CI
Patient count				
N	3,463,831	2,379,586–4,548,069	848,943	521,940–1,175,932
%^a^	12.2%	8.4%–16.0%	3%	1.8%–4.1%
Age, mean	54.5	52.1–57.0	53.7	48.6–58.9
Gender, %				
Male	56.8%	44.2%–69.5%	64.8%	46.6%–82.9%
Female	43.2%	30.5%–55.8%	35.2%	17.1%–53.4%
BMI, mean	37.9	35.7–40.2	37.5	34.9–40.0
BMI category, %				
Underweight (≤18 kg/m^2^)	0.0%	NA	0.0%	NA
Normal weight (18.5–25 kg/m^2^)	1.0%	0%–2.2%	2.5%	1.7%–3.2%
Overweight (25–30 kg/m^2^)	13.6%	7.9%–19.3%	22.4%	12.3%–32.5%
Obese class I (30–35 kg/m^2^)	22.1%	7.9%–36.2%	16.5%	0%–39.3%
Obese classes II and III (≥35 kg/m^2^)	63.3%	46.9%–79.7%	58.6%	35.9%–81.4%
Race				
White	60.4%	47.4%–73.3%	53.5%	35.8%–71.2%
Black	22.9%	13.8%–31.9%	27.0%	16.6%–37.3%
Hispanic	5.9%	1.8%–10.0%	3.9%	0.9%–6.9%
Other	10.8%	5.9%–15.6%	15.7%	1.6%–29.8%
Laboratory values				
Total bilirubin (mg/dL)	0.5	0.4–0.6	0.5	0.4–0.5
AST (IU/L)	29.9	26.9–32.9	42.1	33.6–50.5
ALT (IU/L)	40.0	35.6–44.3	59.6	48.4–70.7
Albumin (g/dL)	4.1	4.0–4.1	4.2	4.0–4.3
Platelet count (10^3^ cells/μL)	244.9	233.9–255.8	242.8	224.0–261.6
HbA1C (%)	7.7	7.3–8.1	7.4	6.8–8.1
Fibroscan values				
LSM (kPa)	11.1	10.3–11.8	13.5	12.5–14.6
CAP (dB)	347.7	335.8–359.6	354.2	340.7–367.7

*Note*: All estimates were weighted to represent the general US population.

^a^ Percentage of eligible patients out of all patients with diabetes in NHANES.

Abbreviations: BMI, body mass index; CAP, controlled attenuation parameter; HbA1c, hemoglobin A1c; LSM, liver stiffness measurement; T2DM, type 2 diabetes mellitus.

## DISCUSSION

In 2017, an estimated 6.65 million adults in the United States were diagnosed with MASH. The lifetime economic burden of managing these patients was projected at $222.6 billion for MASH and $95.4 billion specifically for advanced MASH.^14^,^15^ The predictive analysis estimated an 83% increase in MASH cases, growing from 11.6 million in 2020 to 19.5 million by 2039. This surge is expected to coincide with a 78% rise in cases of advanced liver disease over the same period. Additionally, the cumulative cost per patient for managing MASH is projected to nearly double, increasing from $3636 to $6968, reflecting the parallel rise in advanced liver disease cases.^16^ An analysis from the Global Assessment of the Impact of NASH (GAIN) study, encompassing data from France, Germany, Italy, Spain, the United Kingdom, and the United States, estimated annual costs per patient with MASH at €2763 for direct medical expenses, €4917 for direct nonmedical costs, and €5509 for indirect costs. These findings underscore the substantial global economic burden of MASLD, extending beyond medical expenses to significant nonmedical and indirect costs.^17^ On the other hand, an early cost-effectiveness analysis, based on a willingness-to-pay threshold of $100,000 and considering the reduction in decompensation, liver transplantation, and HCC, estimates that the probability of resmetirom being cost-effective is over 85%.^18^ The results of this study suggest a potential benefit of resmetirom. However, they should be interpreted with caution due to uncertainties in current knowledge and the lack of long-term efficacy data. Moreover, the effect of resmetirom on the prevention of progression to liver cirrhosis or HCC is unknown. Resmetirom is only conditionally approved based on surrogate endpoints, and post-approval data are required to show its clinical benefits.

Our current study—based on weighted analyses—estimated that ~2.4–8.4 million individuals in the general US population might be eligible. In addition, among patients with T2DM, eligibility ranged from 3% to 12%, translating to an estimated 0.85–3.5 million persons. Notably, our findings diverge from those of Fishman et al,^19^ who analyzed the same NHANES 2017–March 2020 cycle but employed a modified NIT strategy to estimate the resmetirom-eligible population. Their analysis estimated that 1699 individuals per one million in the US population could qualify for treatment over a 3-year period.^19^ This divergence underscores the impact of methodological differences, particularly in the use of NIT cutoffs and diagnostic criteria, on patient selection. Such variations can lead to substantial discrepancies in eligibility estimates, highlighting the need for standardization. Our selection criteria resemble those proposed by Noureddin et al^12^ and the 2024 Updates to the AASLD Practice Guidance, with a few exceptions. Due to a lack of data in NHANES, we could not assess MRE, ELF, MASH, or MEFIB. We also applied a higher cutoff for platelet count to be stringent in excluding patients with possible cirrhosis. These differences could lead to both over-estimation and under-estimation of the number of eligible patients. Future research should prioritize harmonizing NITs and their cutoff values to ensure consistency and equity in determining eligibility for resmetirom treatment.

In general, beyond direct clinical benefits, this drug has the potential to substantially reduce the societal burden of MASLD by lowering long-term medical, nonmedical, and indirect costs associated with the disease. Furthermore, equitable access to effective therapies is not only critical for improving public health outcomes but also aligns with the fundamental principle that every individual has the right to receive optimal care without financial hardship. Ensuring fair and widespread access to innovative treatments is essential for advancing health equity and improving quality of life for affected populations.^20^ Making resmetirom available to eligible patients should be a priority for healthcare authorities and policymakers, given its potential to address the progression of MASLD effectively. However, the high cost of the medication poses a significant financial challenge for healthcare systems.^12^

The clinical trials for resmetirom were consistently conducted following liver biopsy, and the impact of prescribing the drug based on the MAESTRO–NASH trial criteria has not yet been evaluated in real-world settings.^10^ This has raised concerns over the potential overuse of the medication following a prescription using NITs. While alternative NITs—including magnetic resonance elastography, FibroScan-AST score, and the Enhanced Liver Fibrosis test—have been suggested as tools for patient evaluation, their practicality and utility require critical assessment, particularly in relation to their availability at different healthcare centers.^12^ In this context, incorporating liver biopsy in carefully selected cases could help alleviate the financial burden while ensuring more accurate identification of the target population for resmetirom therapy. Prescribing resmetirom in the current era, particularly within research settings, represents a promising and prudent approach. On the other hand, results from the MAESTRO–NAFLD trial have shown that the NITs used did not always correspond to at-risk MASH, suggesting a potential overprescription of the drug when relying solely on NITs.^21^ This suggests that the real-world population gaining access to resmetirom may differ from that in the MAESTRO–NASH trial. In the future, optimizing NITs to better identify eligible patients or utilizing liver biopsy in specific cases may prove to be the most effective strategy for maximizing therapeutic precision and resource efficiency.

Until March 2024, lifestyle modifications focused on weight loss were the only available therapeutic option for managing MASLD. However, maintaining long-term weight loss proved challenging for numerous patients, with frequent instances of weight regain—commonly exceeding the initial loss—making sustained disease management increasingly difficult over time.^9^,^22^ Bariatric surgery has demonstrated greater effectiveness in improving liver health compared to lifestyle modifications. However, it is generally reserved for patients with morbid obesity or those with significant obesity-related comorbidities. Although some degree of weight regain is commonly observed by the second postoperative year, the procedure typically results in sustained long-term weight loss. Nevertheless, bariatric surgery carries the risk of serious complications, which must be carefully weighed against its potential benefits when considering treatment options.^23^,^24^ Based on these observations, resmetirom therapy emerges as a safer alternative, given that its adverse effects are predominantly limited to gastrointestinal symptoms, thereby establishing it as a safe treatment option.^10^ Moreover, although resmetirom therapy is associated with a moderate increase in costs, the reduction in expenses related to NASH management underscores its significant cost-effectiveness.^18^,^25^

One interesting finding of our study is that patients with diabetes who were considered eligible for resmetirom had their HbA1c within the treatment target range. This suggests that there is no need to further optimize diabetes control in MASLD patients, either with GLP-1 RAs or other diabetes medications. It also supports the use of a liver centric drug, like resmetirom, in the population with diabetes.

The robustness of our investigation stems from utilizing the NHANES 2017–March 2020 dataset, which provides comprehensive population-level data representative of the United States. This analysis establishes a crucial framework for both healthcare economic planning and public health policy implementation. However, several methodological considerations warrant discussion. Our methodology relied exclusively on FibroScan measurements and laboratory parameters as NITs for determining resmetirom eligibility. While this approach offers practical advantages, these diagnostic tools await full validation and may result in patients being misidentified for treatment. Alternative NITs warrant further consideration in the clinical decision-making process. Given the substantial eligible population identified, liver biopsy remains a critical diagnostic tool in clinical practice to prevent overtreatment and ensure accurate patient selection.

In conclusion, our findings indicate that ~8.4 million individuals in the United States meet the eligibility criteria for resmetirom therapy. This significant treatment-eligible population underscores the need for rigorous patient selection protocols to optimize cost-effectiveness, integration of resmetirom into comprehensive healthcare strategies, and standardization of diagnostic approaches. These insights provide valuable guidance for healthcare stakeholders in resource allocation and therapeutic implementation strategies, particularly as we advance toward more precise and cost-effective treatment paradigms in MASH management.
